# Early life adversity diminishes the cortisol response to opioid blockade in women: Studies from the Family Health Patterns project

**DOI:** 10.1371/journal.pone.0205723

**Published:** 2018-10-12

**Authors:** William R. Lovallo, Ashley Acheson, Andrea S. Vincent, Kristen H. Sorocco, Andrew J. Cohoon

**Affiliations:** 1 Behavioral Sciences Laboratories, Veterans Affairs Medical Center, Oklahoma City, OK, United States of America; 2 Department of Psychiatry and Behavioral Sciences, University of Oklahoma Health Sciences Center, Oklahoma City, OK, United States of America; 3 Department of Psychiatry, University of Arkansas Medical Center, Little Rock, AK, United States of America; 4 Cognitive Science Research Center, University of Oklahoma, Norman, OK, United States of America; 5 Donald W. Reynolds Department of Geriatric Medicine, University of Oklahoma Health Sciences Center, Oklahoma City, OK, United States of America; National Institue on Drug Abuse, UNITED STATES

## Abstract

Early life adversity (ELA) contributes to behavioral impulsivity along with risk for substance use disorders, both accompanied by blunted stress-axis reactivity. However, the biological contributors to blunted stress reactivity are not known. We took advantage of the fact that women have significant opioid inhibition of cortisol output by using the opioid antagonist, naltrexone, to unmask opioid interactions due to ELA. We administered 50 mg of naltrexone or placebo to 72 healthy women (23 years of age) in a double-blind crossover study and observed deviations in cortisol secretion from placebo over the next 180 minutes. ELA was assessed by reported exposure to physical and sexual abuse or neglect and low socioeconomic status and scored as Low, Medium, or High (0, 1–2, and 3+). The ELA groups all had identical placebo-day cortisol secretion, indicating normal basal regulation of the hypothalamic-pituitary-adrenocortical axis. Cortisol rises to naltrexone were largest in the Low-ELA group and strongly blunted in the High-ELA group (*F* = 3.51, *p* = 0.035), indicating a lack of opioid function in women with high degrees of ELA. The Low-ELA women reported dysphoric responses to naltrexone (*F* = 4.05, *p* = .022) indicating a mild opioid withdrawal, an effect that was absent in the High-ELA group. Women exposed to ELA have blunted cortisol responses to naltrexone, indicating reduced opioid regulation of the stress axis. Central opioid changes may be one pathway linking ELA to blunted stress reactivity in adulthood.

## Introduction

Blunted cortisol responses to stress are associated with negative health outcomes that include risky behavior, abusive intake of alcohol, and experimentation with drugs [1]. As such, blunted stress reactivity may constitute a risk marker or a risk factor for substance use disorders (SUD). This relationship raises two questions: What causes blunted stress reactivity to develop in some individuals? When blunted stress reactivity is present, how does it contribute to risky behavior and SUD? Answers to these questions may contribute to a mechanistic understanding of SUD risk.

A recent body of evidence shows that blunted reactivity occurs in persons who have experienced early life adversity (ELA), usually assessed by a reported history of physical and sexual abuse and neglect [2, 3], and also by low socioeconomic status [4]. However, the relationship between ELA and blunted reactivity presently lacks a known physiological mechanism. Although ELA is likely to modify brain systems that respond to external events, such mechanisms are only beginning to be explored [5–7]. In the present analysis, we used the opioid antagonist naltrexone to probe reactivity of the central opioid system in women with differing histories of ELA exposure.

Opioid agonists and antagonists exert opposing effects on the hypothalamic-pituitary-adrenocortical axis in humans [8, 9]. Cortisol secretion is inhibited by opioid agonists and increased by opioid antagonists [10–12]. Accordingly, the size of the cortisol response to opioid blockade provides a useful probe for individual differences in underlying opioid regulation, including risk for SUD [12, 13]. Using similar logic, we showed that women, compared to men, had smaller cortisol responses to stress but larger responses to naltrexone blockade [14]. This yin-yang relationship suggested that women have a greater level of tonic activity in the central opioid system that diminishes cortisol responses to stress but increases them to naltrexone antagonism [14–16]. This apparent sex difference in opioid regulation was reinforced by the finding that female carriers of the high-affinity, G-allele of the μ-opioid receptor gene, *OPRM1*, had a complete absence of stress response in the laboratory [17], an effect that was not seen in men. The high level of intrinsic opioid activity in women thereby provided an opportunity to test for an opioid contribution to blunted cortisol reactivity in women exposed to ELA. We therefore examined the salivary cortisol response to naltrexone (50 mg) in women with differing histories of ELA. We reasoned that women exposed to high levels of ELA should have altered cortisol responses to naltrexone blockade.

## Materials and methods

This project was reviewed and approved by the Institutional Review Board of the University of Oklahoma Health Sciences Center and the VA Medical Center. Project number 2302. All volunteers signed an informed consent form.

### Overview

The Oklahoma Family Health Patterns Project is a study of healthy young adults with the goal of understanding preexisting differences that might provide insights into risk factors for alcoholism. The project has identified ELA exposure as a significant contributor to personal, behavioral, and stress reactive characteristics that may contribute to alcoholism. We tested the effect of naltrexone on a subset of persons raking part in this larger project.

### Subjects

Naltrexone testing included 76 females recruited through community advertisement, however 4 women experienced nausea and vomiting in response to naltrexone and discontinued the protocol, and so the final sample includes 72 women. Each subject signed a consent form approved by the Institutional Review Board of the University of Oklahoma Health Sciences Center and the Veterans Affairs Medical Center, Oklahoma City, OK, USA, and received financial compensation for participating.

#### Inclusion and exclusion criteria

Prospective volunteers were excluded if they had: a history of any Axis I disorder (other than past depression, > 60 days prior), alcohol or drug dependence, or met criteria for any substance abuse within the past 2 mo, as defined by the Diagnostic and Statistical Manual of Mental disorders, 4^th^ ed. [18], or provided a positive urine drug screen (iCup, Instant Technologies, Norfolk VA) or breath alcohol test on days of testing. Women were required to have a negative urine pregnancy test on each day of testing. All participants were in good physical health, between the ages of 18 and 30 yr, had a body mass index 18.5–29 kg/m^2^, were not taking prescription medications other than hormonal contraceptives, and had no reported history of serious medical disorder. Smoking and smokeless tobacco use were not exclusionary. Smokers were allowed one cigarette immediately prior to the start of the protocol to minimize cravings during testing. Alcohol consumption was assessed using a quantity-frequency index, and hazardous drinking practices were assessed using the Alcohol Use Disorders Identification Test [19].

#### Assessment of early life adversity

Early stress exposure and low SES are associated with a wide range of negative health outcomes [20] including SUD [21]. ELA scores were derived during the clinical interview from items on the posttraumatic stress disorders module on the C-DIS-IV, which has a high degree of test-retest and interinstrument reliability [22]. None of the subjects met full diagnostic criteria for PTSD. The items used for ELA assessment are closely similar to the life events assessed retrospectively in the studies by Caspi [23] as follows: Physical or Sexual Adversity (Have you ever been mugged or threatened with a weapon or ever experienced a break-in or robbery? Have you ever been raped or sexually assaulted by a relative? Have you ever been raped or sexually assaulted by someone not related to you?), and Emotional Adversity (Before you were 15, was there a time when you did not live with your biological mother for at least 6 months? Before you were 15, was there a time when you did not live with your biological father for at least 6 months?). ELA scores from the interview items ranged from 0 (no adverse events) to 5 events.

SES was calculated using Hollingshead and Redlich’s system, based on the highest occupational level attained by the primary breadwinner of the subject’s childhood household [24].

Composite ELA scores ranging from 0 (no adverse events) to 5, plus SES scores falling into the upper (0), middle (1), and lower (2) third of the distribution for our subject population, yielded composite ELA scores ranging from 0–8. These composite scores were then recoded as 0, 1–2, and 3+ for analysis.

### Study design and procedure

The study used a randomized, placebo-controlled, double-blind administration of placebo vs. oral naltrexone (50 mg, Malinkrodt, St. Louis, MO, USA) in identical appearing capsules prepared by a compounding pharmacy (Innovative Pharmacy Solutions, Edmond, OK, USA). Test days were separated by a minimum of 72 hrs. Naltrexone is a competitive opioid receptor antagonist with a long duration of action at central opioid receptor sites [25], and is approved in treatment of alcohol and opioid dependencies.

The subject entered the General Clinical Research Center at the University of Oklahoma Health Sciences Center at 0800h, provided a urine sample to check for the presence of opiates or other drugs, and was served a light breakfast. At 0900h he or she provided a baseline saliva sample and immediately consumed the naltrexone or placebo capsule. Saliva was then collected every 30 min for the next 180 min. Every 60 min, the subject filled out an opioid-specific side effects questionnaire [12] and also rated their moods using visual-analogue scales [26]. The subject remained seated in a recliner chair through the entire protocol and read general interest magazines or watched videos of nature or history programs. A research nurse monitored subjects during the protocol.

### Saliva collection times and cortisol assay

Saliva samples were collected using the Salivette device (Sarstedt, Newton, NC, USA).

Salivettes were centrifuged at 4200 RPM for 20 min. The saliva was transferred to cryogenic storage tubes and placed into a– 20º C freezer until shipping. Saliva free cortisol assays were conducted by Salimetrics (State College, PA, USA) using a competitive enzymatic immunoassay [27] with a sensitivity of < .083 μg/dL and an interassay coefficient of variation of < 6.42%.

Estrogen levels may affect cortisol binding. Preliminary analyses compared cortisol responses in women during the luteal and follicular phases of the menstrual cycle and in women using hormonal birth control (35%). Neither hormonal influence affected cortisol responses *(t*s ≤ 0.55, *p*s ≥ 0.58). Menstrual cycle and hormonal contraceptive effects were therefore not considered in subsequent analyses.

### Subjective responses and side effects

Subjects rated their subjective states at four time points (0, 60, 120, and 180 min) using 12 10-point visual-analogue scales containing a Distress subscale (impatience, irritability, distress, pleasantness, and control), and an Activation subscale (effort, tension, concentration, interest, and stimulation) adapted from Foresman [28].

Side effects to naltrexone were assessed at two time points (0 and 180 min) using an opioid-specific questionnaire [12] having 3-point scales (none, mild, or severe) covering nausea, vomiting, headache, distress, warm or flushed feelings, anxiety, libido, hives or rash, insomnia, diarrhea, pain, sleepiness and agitation. Four subjects vomited and were excused from the protocol and monitored by the nursing staff. Other than mild nausea, there was a paucity of side effects reported by the remaining women, and adverse effects were therefore not considered to influence the cortisol responses examined here.

### Data analyses

Demographic and biometric characteristics were compared between the female ELA groups using *F*-ratios for continuous variables and an exact chi-square test for categorical variables. The cortisol response to naltrexone in the 0, 1–2, and 3+ ELA groups was tested in a 1-way ANOVA using the mean of the difference scores from placebo to naltrexone day over the period of maximum response (90, 120, and 150 minutes postdosing). Subjective reports of Activation and Distress were examined in Day x Period (0, 60, 120, 180 min) ANOVAs. Significance level was set at α < 0.05. Data were analyzed using SAS software, Ver. 9.2 for Windows. Copyright 2012 SAS Institute Inc. SAS and all other SAS Institute Inc. product or service names are registered trademarks of SAS Institute Inc., Cary, NC, USA.

## Results and discussion

Table 1 presents demographic and biomorphic characteristics of the ELA groups. Since a focus of the parent study was to assess drinking and drug use, the groups were also compared on alcohol intake and smoking. The ELA groups were not different in any of these variables.

**Table 1:** Demographic and biomorphic characteristics of ELA groups.

	Early Life Adversity Score			
	0	1–2	3+	p-value
**N**	12	44	16	
**Age**	23.9 [0.6]	23.8 [0.4]	21.1 [0.7]	0.36
**Race (% White)**	100	91	94	0.99
**Education (yr)**	16.9 [0.4]	15.7 [0.3]	15.4 [0.5]	0.19
**Body mass ind.**	22.2 [1.0]	23.0 [0.4]	22.3 [0.8]	0.23
**Smokers (%)(n)**	8.3 (1)	6.8 (3)	0 (0)	0.62
**AUDIT**^**a**^	4.67 [0.8]	3.77 [0.3]	3.12 [0.7]	0.18
**QFI**^**b**^	19.7 [4.6]	17.9 [2.8]	18.7 [6.9]	0.44

Entries show M ± SEM unless noted otherwise. P-values are for F tests on continuous variables and Chi square tests for the categorical variables, race, and percentage of smokers.

^a^ Alcohol Use Disorders Identification Test

^b^ Quantity-frequency index of alcohol consumption in oz of pure ethanol mo

### Cortisol responses to naltrexone

**Fig 1:**
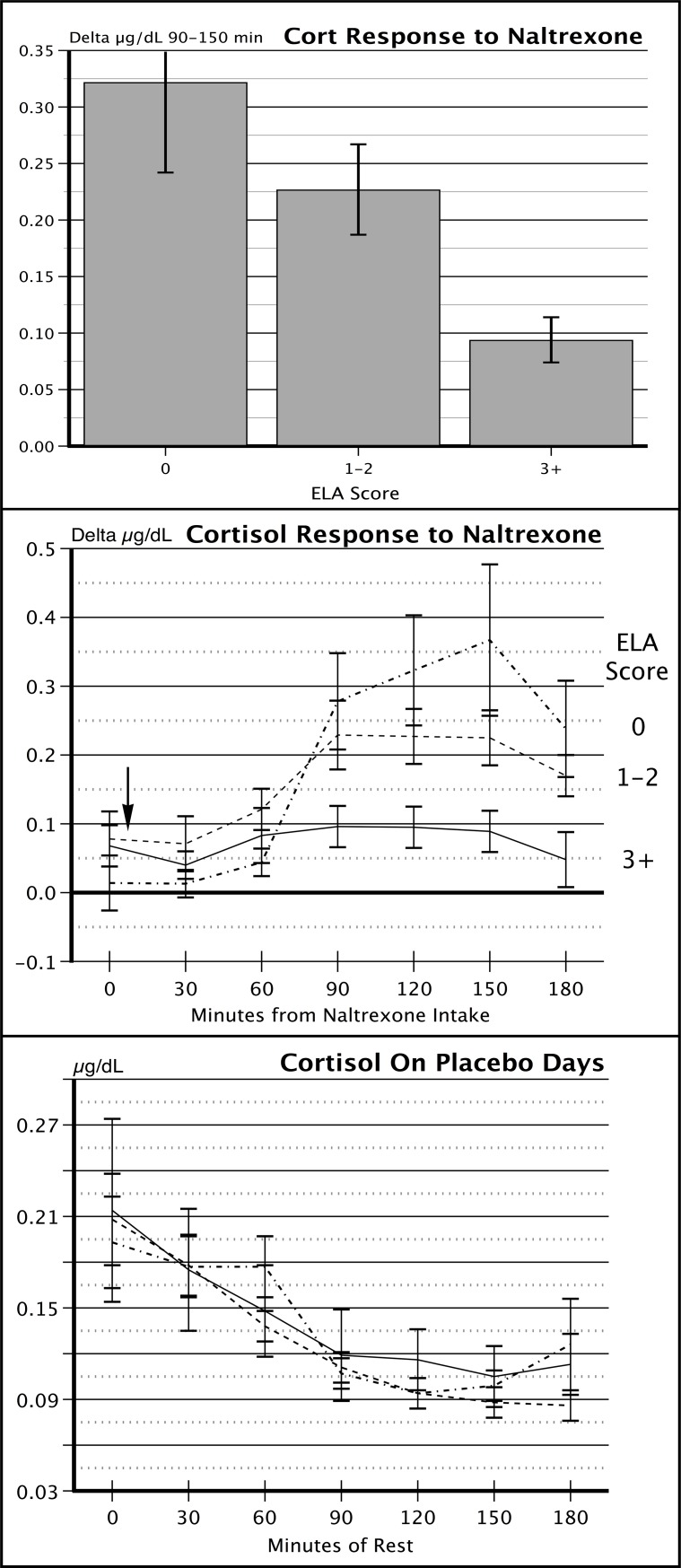
Saliva cortisol values in response to naltrexone in women with Low (0), Medium (1–2), and High (3+) levels of Early Life Adversity. Top panel: change in saliva cortisol from the placebo day to the naltrexone day averaged over 90–150 minutes after administration of 50 mg naltrexone or placebo. Middle panel: change in saliva cortisol on naltrexone day at each collection interval. Arrow indicates time of naltrexone or placebo administration. Bottom panel: Saliva cortisol on placebo day for the three ELA groups.

Cortisol responses to naltrexone for the three ELA groups are shown in Fig 1, top panels. The response pattern shows a dose-response effect of ELA on cortisol reactivity (*F* = 3.51, *p* = 0.035) with the largest responses in the 0 ELA group and the smallest in the 3+ group. Fig 1, bottom panel, shows that the ELA groups had nearly identical resting cortisol values during the morning hours, indicating that the blunted cortisol responses in the 3+ ELA group were not a function of disturbed basal function. The middle panel of Fig 1 indicates that the ELA groups started the protocol with nearly identical baseline cortisol secretion levels. The 3+ ELA group had no significant variation in cortisol values over time, indicative of an absence of opioid influence, while the 0 ELA group had a substantial increase in cortisol, consistent with an acute withdrawal of central opioid restraint over the hypothalamic-pituitary-adrenocortical axis. Due to reported nausea in some women, the ANOVA on cortisol values was rerun with nausea as a covariate, and the results did not change.

### Subjective responses to naltrexone

Naltrexone blockade is expected to diminish the calming effect of endogenous opioids and result in a sense of dysphoria. As shown in Table 2, naltrexone was associated with increased reports of Distress (*F* = 14.13, *p* = .0004). This main effect was qualified by a significant naltrexone by ELA interaction (*F* = 4.13, *p* = .020). In this case, women with 0 ELA showed the expected increase in reports of Distress on the naltrexone day consistent with an acute opioid withdrawal. In contrast, women in the 3+ ELA group reported no such change, consistent with a low level of endogenous opioid tone. Reports on the Activation scale showed no significant change across days for any of the ELA groups (Fs ≤ 1.19, ps ≥ .278).

**Table 2:** Reports of activation and distress following naltrexone.

ELA group	0	1–2	3+
**Distress**			
**Placebo**	2.21 [0.3]	2.13 [0.1]	2.18 [0.3]
**Naltrexone**	2.76 [0.3]	2.32 [0.1]	2.16 [0.3]
**Activation**			
**Placebo**	1.96 [0.3]	2.04 [0.2]	2.59 [0.5]
**Naltrexone**	1.73 [0.3]	2.08 [0.2]	2.48 [0.4]

Entries show M ± SEM. Entries are means of the reported levels of distress (mean of impatience, irritability, distress, pleasantness, and control) and activation (mean of effort, tension, concentration, interest, and stimulation) recorded at 60, 90, and 120 minutes post administration of placebo or naltrexone.

We tested women with differing histories of ELA exposure and used the cortisol response to naltrexone blockade to measure differences in opioid-mediated reactivity of the hypothalamic-pituitary-adrenocortical axis. Cortisol responses to 50 mg naltrexone were greatest for women who reported little or no history of life stress prior to age 15 (0 ELA) and smallest for women reporting more substantial levels of adversity (3+ ELA). Women in the 0 ELA group also reported an acute sense of distress following naltrexone, a subjective effect that was absent in the 3+ ELA group. Both of these findings are consistent with acute withdrawal of endogenous opioid function following naltrexone blockade that was larger in the 0 ELA group and smaller in the 3+ ELA women. This suggests that higher levels of ELA may diminish endogenous opioid activity in adulthood, thereby reducing any residual effect of its removal by naltrexone blockade in the 3+ women. The finding of an ELA effect operating through the central opioid system compliments earlier studies of diminished cortisol reactivity, and it suggests avenues for future research on central opioid pathways in relation to psychological and behavioral influences on health behaviors in adulthood.

A growing body of data points to blunted stress axis reactivity in persons who have been exposed to ELA [1]. In turn, both ELA and blunted stress reactivity are associated with a wide range of poor health outcomes, in particular with alcohol and other substance use disorders [29–31]. Despite this associational evidence, there is a lack of mechanistic information connecting these adverse events in early life with their physiological and behavioral outcomes in adulthood. Information about such mechanisms could contribute to an understanding of risk factors for alcohol and other substance use disorders. The present data are consistent with an interpretation in which women with little ELA history have normal levels of opioid function that are progressively diminished by greater levels of ELA exposure to childhood adversity. Since endogenous opioids contribute to regulation of moods and responses to stressful events, diminished opioid activity in the High ELA women may help direct research to related central neuromodulator systems.

The finding that ELA predicts reduced cortisol response to naltrexone parallels other data showing that ELA leads to diminished cortisol reactivity to psychological stress in healthy young adults [3]. The cortisol response blunting to naltrexone reported here extends this finding from psychological stressor challenge to pharmacologic blockade. This points to a significant and pervasive effect of early stress exposure on central mechanisms regulating the stress response. However, the present results do not appear to reflect a global, nonspecific loss of HPA function. First, our measurements of cortisol response in the placebo condition point to normal levels of adrenal output and comparable changes over the daytime hours in the ELA groups, indicative of normal diurnal, metabolic, and feedback regulation of the HPA. This result matches our report in a larger study sample [3]. Second, ELA diminishes both cortisol and heart rate responses to psychological stress [3]. The present analysis relied on resting measures of cortisol secretion, a time when heart rate levels were stable and unreactive, and therefore no comparison of cortisol and heart rate responses was possible [32]. Third, the changes in subjective state following naltrexone were greatest in the 0 ELA subjects, the group that presumably had the greatest withdrawal of basal central opioid function following naltrexone, and least in the 3+ ELA group. These observations indicate that blunted reactivity does not arise from any intrinsic dysfunction of the HPA. Instead, the effects may indicate a specific adaptation of the central opioid system to early experience that shapes how persons respond to the environment, and in particular to motivationally significant stimuli.

The present analysis was confined to women since opioid regulation of the stress axis appears to be very minor in males [14, 17]. Women exposed to naltrexone have greater antinociceptive and sedative responses than men do [33, 34]. In a previous paper, we showed that this dose of naltrexone produced a very robust cortisol response in women and a negligible change in males [14]. Similarly, genetic polymorphisms affecting opioid regulation are greater in women and smaller in males; women show diminished stress response if they have the high-affinity G-allele of OPRM1 relative to homozygous carriers of the low affinity allele (AA) [17]. This genotype had no influence on stress responses in males. Despite these sex differences in opioid effects, we have shown that ELA exposure results in blunted stress reactivity in both men and women [3]. Accordingly, we view the present opioid findings as one potential contributor to the impact of ELA on the stress axis, although it calls attention to effects of ELA in women and provides less information on these processes in males.

The normal levels of basal cortisol secretion in the three ELA groups tend to eliminate from consideration some potential sources of blunted stress reactivity. The diurnal secretion of cortisol has been well characterized in terms of circadian clock genes in the suprachiasmatic nucleus of the hypothalamus along with inputs from the pineal gland that together coordinate signals to the hypothalamic paraventricular nucleus and median eminence [35]. These signals regulate output of corticotropin releasing factor to the pituitary and adrenocorticotropin to the adrenal cortex, with negative feedback by cortisol at the hypothalamus and pituitary. This circadian rhythm is essential for normal entrainment of gene expression in peripheral tissues. The diurnal pattern of cortisol secretion, adrenal cortex activity, and negative feedback within the HPA are apparently unaffected in persons with high levels of ELA exposure. Accordingly, diminished cortisol response to naltrexone blockade does not appear to reflect any intrinsic modification of HPA regulation. However, during periods of threat, a second system of inputs originating from the amygdala act on neurons of the paraventricular nucleus and the brainstem locus ceruleus to increase generalized arousal via a distributed network of norepinephrine synthesizing neurons. In addition, the paraventricular nucleus has a second set of corticotropin releasing factor—arginine vasopressin secreting neurons that generate stress levels of adrenocorticotropin secretion at the pituitary [36]. It is possible that exposure to ELA could modify signaling at this level in the system resulting in blunted reactivity to stress. The central opioid system regulates activity at the hypothalamus and brainstem locus ceruleus, and attenuated opioid function at these structures is of potential interest in exploring the impact of ELA in women [37, 38].

There is a small literature that bears on the present topic. A number of recent neuroimaging studies have sought to examine brain mechanisms controlling stress responses and how the impact of childhood adversity may modify these systems [39]. These have revealed that reactions to emotionally relevant stimuli engage communication between the amygdala and the region of convergence of the orbitofrontal cortex, the anterior cingulate cortex, and ventromedial prefrontal cortex [40–42]. This entire network is densely supplied with a wide range of neuromodulators including neurons of the central corticotropin releasing factor system [43] and the central opioid system, among others. Childhood maltreatment and upbringing in a disadvantaged household may produce lasting changes in the way these areas of the brain respond to emotional stimuli and stressful tasks [44–46].

We have approached the present results as reflecting a specific deficit of opioid signaling to the stress-sensitive glucocorticoid response in women who had higher levels of ELA exposure. This interpretation implies that direct linkages between opioid function and glucocorticoid regulation are impaired by a history of ELA, accounting for the present results. A second possibility is that the stress-reactive component of the cortisol response is blunted in high ELA women for reasons other than strict opioid regulation. In this case the central opioid system may be functioning normally but opioid blockade may fail to activate downstream nonopioid mechanisms activating the stress axis. Under this scenario, diurnal and metabolic activity of the HPA would appear normal, but stress related activation would be blunted relative to any given agonist. Additional study of ELA-exposed women is therefore warranted to examine these two possibilities.

We tested only a single dose of naltrexone, raising the possibility that the women may have had a larger weight-adjusted dose than men. However, other studies using 50 and 100 mg of naltrexone and doses of naloxone as high as 400 μg/kg and consistently found larger cortisol responses in women than in men [15, 16, 47]. Accordingly, it is less likely that the present dose of naltrexone underestimated the effect of opioid regulation in men.

Although we have reported blunted stress responses in women exposed to ELA, others have reported exaggerated responses in traumatized women with depression [48]. The present results are confined to healthy women; the entry criteria in the Family Health Patterns project were targeted to healthy individuals who are free of psychiatric morbidity and a history of trauma. Exclusion criteria ensured that none of the women in the present sample met criteria for posttraumatic stress disorder. Accordingly, the present data do not provide insight into HPA dysfunction in traumatized persons [48]. Although the results are limited to healthy nontraumatized women, the impact of ELA and the presence of blunted stress reactivity point to the pervasive effects of early experience.

The present sample of 70 women limits our ability to evaluate how modified cortisol responses to naltrexone are related to a range of risk factors for SUD. Future studies on a larger sample may provide additional evidence on the possible role for diminished opioid function in SUD and risky behaviors. Finally, although cortisol reactions are modified by phase of the menstrual cycle and the use of hormonal contraceptives, neither of these factors influenced the present findings.

## Conclusions

Early life adversity may downregulate the effects of the endogenous opioid system in women. The opioid antagonist, naltrexone normally produces a robust cortisol response in women, but this expected response was progressively diminished in nontraumatized women who reported greater levels of adversity in childhood and early adolescence. Increasing evidence shows that blunted stress axis reactivity is associated with altered emotional reactivity, impulsive behaviors, and risk for substance use disorders. The central opioid system may therefore be a pathway through which early experience modifies responses to the environment in adulthood., potentially influencing health behaviors.
